# Deposition of silica in sorghum root endodermis modifies the chemistry of associated lignin

**DOI:** 10.3389/fpls.2024.1370479

**Published:** 2024-04-03

**Authors:** Nerya Zexer, Sabrina Diehn, Rivka Elbaum

**Affiliations:** ^1^ Department of Biology, The Pennsylvania State University, University Park, PA, United States; ^2^ The Robert H. Smith Institute of Plant Sciences and Genetics in Agriculture, The Hebrew University of Jerusalem, Rehovot, Israel

**Keywords:** ITCW, lignin, Raman microspectroscopy, root endodermis, silicic acid, silicon, sorghum

## Abstract

Silica aggregates at the endodermis of sorghum roots. Aggregation follows a spotted pattern of locally deposited lignin at the inner tangential cell walls. Autofluorescence microscopy suggests that non-silicified (-Si) lignin spots are composed of two distinct concentric regions of varied composition. To highlight variations in lignin chemistry, we used Raman microspectroscopy to map the endodermal cell wall and silica aggregation sites in sorghum roots grown hydroponically with or without Si amendment. In +Si samples, the aggregate center was characterized by typical lignin monomer bands surrounded by lignin with a low level of polymerization. Farther from the spot, polysaccharide concentration increased and soluble silicic acid was detected in addition to silica bands. In -Si samples, the main band at the spot center was assigned to lignin radicals and highly polymerized lignin. Both +Si and -Si loci were enriched by aromatic carbonyls. We propose that at silica aggregation sites, carbonyl rich lignin monomers are locally exported to the apoplast. These monomers are radicalized and polymerized into short lignin polymers. In the presence of silicic acid, bonds typically involved in lignin extension, bind to silanols and nucleate silica aggregates near the monomer extrusion loci. This process inhibits further polymerization of lignin. In -Si samples, the monomers diffuse farther in the wall and crosslink with cell wall polymers, forming a ring of dense lignified cell wall around their export sites.

## Introduction

Plant cell walls are made of cellulose which is crosslinked by hemicellulose, pectin and lignin. In grasses, in addition to monolignol, high concentration of ferulic acid is incorporated in the lignin, allowing for lignin crosslinking to hemicellulose (Hatfield et al., 2017). Silica may add to the cell wall structure in some plant groups, and most pronouncedly the grasses family (Hodson et al., 2005). Silica is suggested to be a cost-effective substitution for lignin, with a similar stiffening effect of the wall (Hodson and Guppy, 2022). Supporting this hypothesis, lignin and silica are negatively correlated in some plants (Schoelynck et al., 2010; Zhang et al., 2015; Rivai et al., 2022). Silica is also negatively correlated to leaf longevity, suggesting silica as an economical building block of the cell wall with some disadvantages of being heavier per volume (denser) and less inclined to modifications, in relation to the more costly lignin (Cooke and Leishman, 2011). In contrast, some studies suggest silica and lignin may deposit together (Pan et al., 2017; Zexer and Elbaum, 2020).

**Figure 1:**
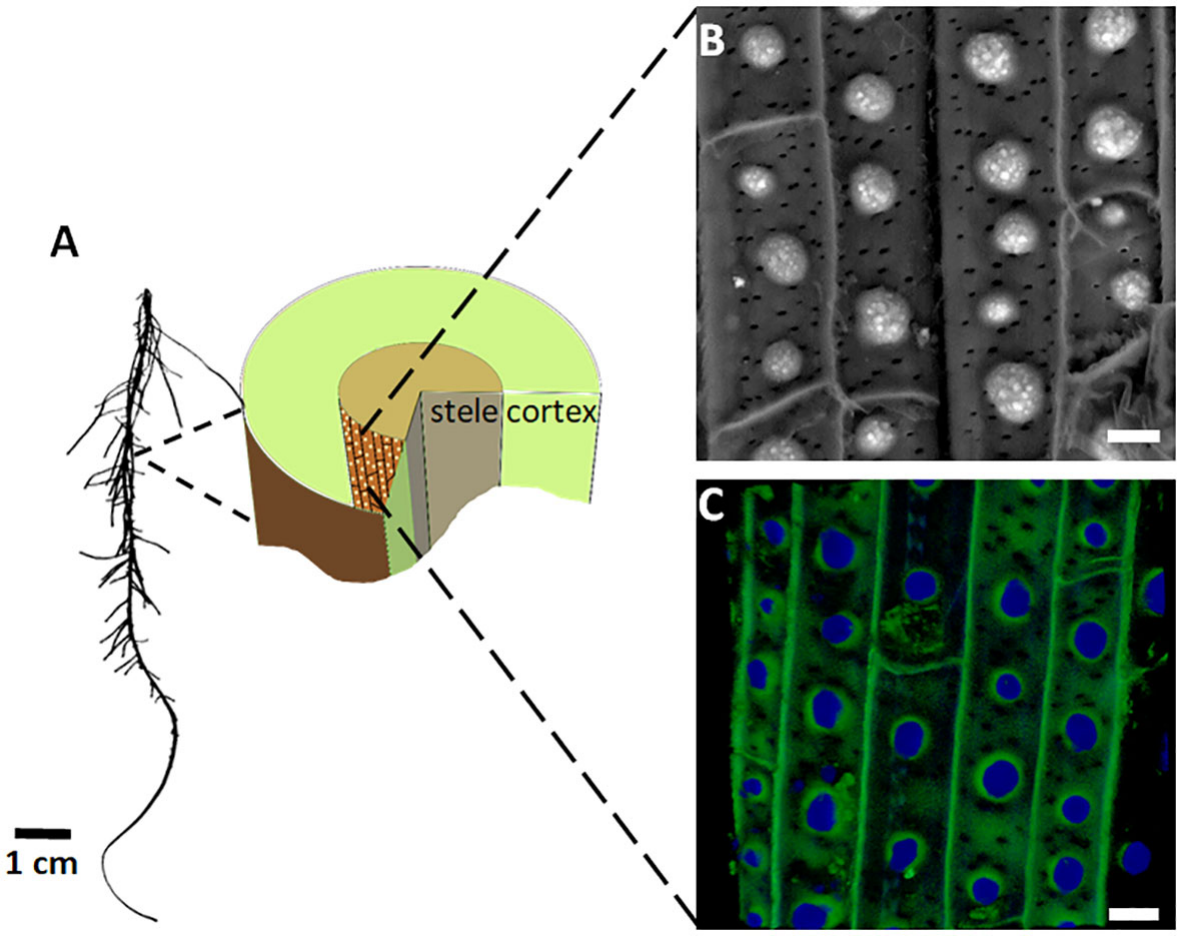
Silica and lignin deposition at the endodermis of sorghum roots. **(A)** Image of a sorghum seedling root (left) and a diagram of its cross section (right). The endodermis cell layer, which separates the cortex and stele, is colored brown. Bright dots on the endodermis inner tangential cell wall (ITCW) mark the lignin and silica deposits. **(B)** SEM image in the back-scattered electron mode of the endodermis after removal of the cortex. Silica aggregates appear white. **(C)** Typical autofluorescence of silicified endodermis, showing the silica aggregates in blue and the ITCW in green. Scale bars in panels **(B, C)**, represent 10 μm. Modified from Zexer and Elbaum (2020).

Silica aggregation in the endodermis of sorghum roots follows an ordered array of spots made of specialized lignin (**Figure 1**). Silicic acid condensation into silica occurs only in newly synthesized cell walls (Soukup et al., 2020), suggesting a specific moiety that can react with silicic acid for a limited time. Elimination of the lignin deposition is possible via the activity of potassium iodide (KI) that quenches hydrogen peroxide and the radicalization of lignin monomers. Under KI treatment silica does not aggregate in the endodermis, suggesting that silicic acid condenses onto cell walls containing the specialized lignin (Zexer and Elbaum, 2022). The spots of specialized lignin appear blue under autofluorescence only when they are silicified. When silicic acid is not available during the deposition of the specialized lignin, its autofluorescence shifts from blue to green (Zexer and Elbaum, 2020).

**Figure 2:**
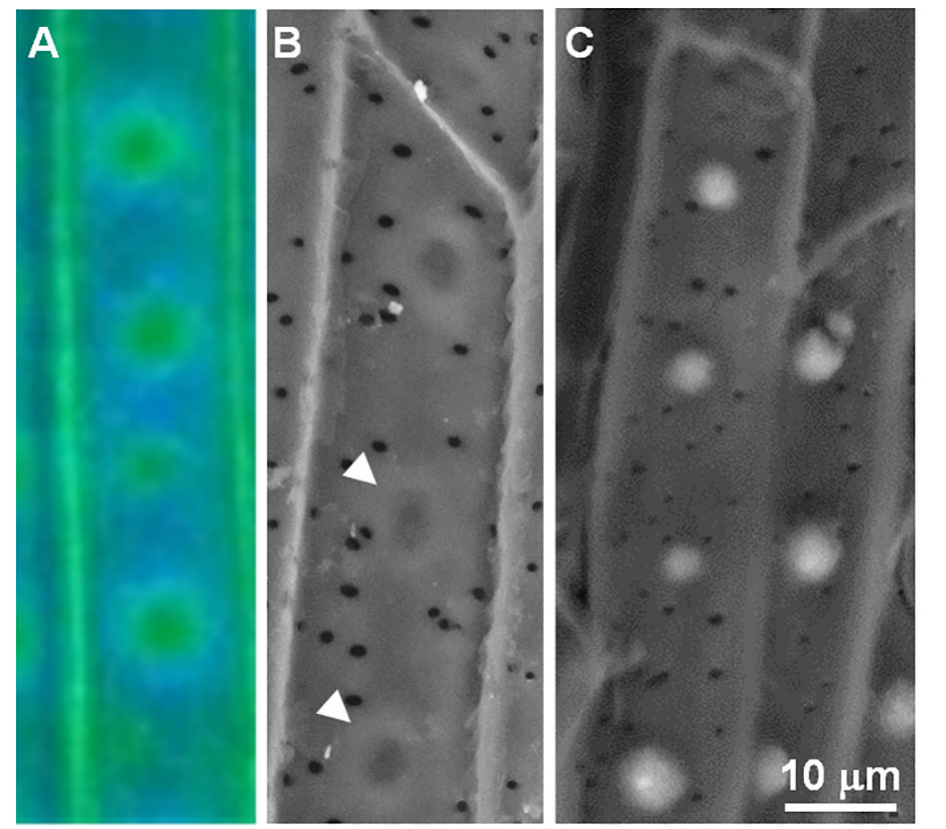
Autofluorescence and of the endodermal ITCW suggesting concentric diffusion of cell wall monomers in -Si walls. **(A)** Autofluorescence at pH 12 and **(B)** back scattered electron micrograph demonstrating the doughnut-shaped cell wall in –Si ITCW (arrowheads). **(C)** Back scattered electron micrograph of +Si ITCW. The dense doughnut-shaped cell wall does not form in the presence of silicic acid. Scale bar common to SEM micrographs represents 10 μm. Modified from (Zexer and Elbaum, 2020).

In order to form a spot of specific cell wall composition, monolignols and other cell wall precursors should be exported from a small location on the plasma membrane. While some lignification processes may rely on monolignols from neighboring cells (Smith et al, 2013), the localized lignin formation close to the cell membrane does not support such a process. The monolignols may then diffuse away from their exporting site and interact with apoplastic silicic acid to form circular silica aggregation. In the absence of silicic acid, the monolignols polymerize and bind to the wall. Autofluorescence and SEM images suggest that the monolignols take time to react with the existing cell wall, and thus diffuse for about 2 - 4 μm before binding into a highly crosslinked doughnut-shaped structure (**Figure 2**). Interestingly, ferulic acid oxidative coupling may crosslink the wall and also change its fluorescent properties as it binds either to hemicellulose or lignin (Ishii, 1997).

The autofluorescence of a -Si spot at high and neutral pH values reveals a radial structure of the lignified spot (Zexer and Elbaum, 2020). The center shows stronger green fluorescence under neutral pH than at pH 12. A doughnut shaped structure surrounding the center is blue at neutral pH and green at high pH values. Such changes in the autofluorescence may indicate ferulic acid bound to hemicellulose by an ester bond (Harris and Hartley, 1976; Abraham et al., 2018). At locations farther from the spot center, blue autofluorescence at neutral pH intensifies at pH 12, which may indicate non-modified lignin (Abraham et al., 2018).

To illuminate chemical aspects of lignification at silica deposition sites, we collected Raman maps from the endodermis of sorghum roots grown hydroponically with or without silicic acid supplementation. To avoid variation caused by growing conditions and age we examined aggregates in the primary root of one week old seedlings, in a root region where aggregates reached full size and there is no formation of new aggregates. Our results show that in -Si roots, lignin spots are divided to concentric regions with minor spectral variations representing highly polymerized lignin and lignin radicals. In +Si samples we identified lower levels of lignin polymerization and lignin monomers trapped in the silica. Carbonyls are typical to both +Si and -Si, and may characterize silica depositing tissues.

## Results and discussion

**Figure 3:**
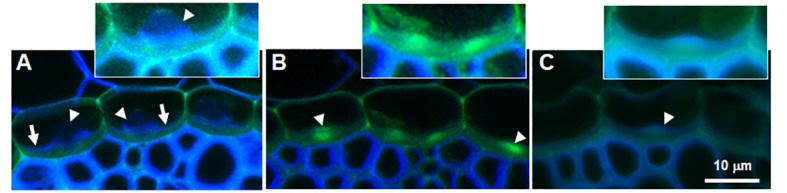
Autofluorescence of cross sections of sorghum root endodermis. **(A)** Root grown with silicic acid showing blue autofluorescence at the base of silica aggregates (arrowheads) and green autofluorescence at the ITCW (arrows). **(B)** Root grown without silicic acid showing a bright green fluorescence spot at the ITCW (arrowheads). **(C)** Root grown without silicic acid and then transplanted into a fresh solution supplemented with silicic acid for 3 hours. New cell wall with blue autofluorescence deposited on the green ITCW (arrowhead). Scale bar represents 10 μm in main panels. Insets show magnified ITCW of one endodermis cell with increased brightness.

Confocal fluorescence microscopy of the endodermis inner tangential cell wall (ITCW) of sorghum roots grown with silicic acid (+Si) revealed the 3D structure of the lignin spots. Concave region with green autofluorescence delineated silica aggregates fluorescing in blue (**Supplementary Video S1**). In roots grown without supplemental silicic acid (-Si), locations of potential silica aggregation showed green encircled by blue autofluorescence (**Supplementary Video S2**). To understand whether the change in autofluorescence was due to binding of silicic acid, we examined cross sections of -Si roots that were exposed to +Si medium for 3 hours. Blue autofluorescence region appeared on top of a green fluorescent region (**Figure 3**). The newly deposited cell wall was possibly silicified, as silica aggregates form as soon as 2 hours after exposure to silicic acid (Zexer and Elbaum, 2020).

**Figure 4:**
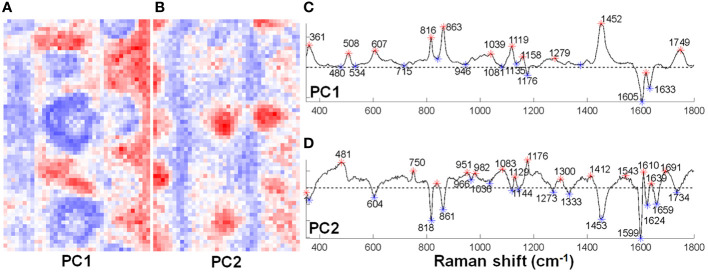
Principal component analysis (PCA) of two Raman maps of sorghum root ITCW grown with silicic acid. The distribution of positive (red) and negative (blue) score values for **(A)** PC1 and **(B)** PC2. **(C)** PC1 and **(D)** PC2 corresponding loadings with asterisks highlighting positive (red) and negative (blue) score values. Positive PC1 bands and negative PC2 were assigned to polysaccharides (361, 818, 861, and 1453 cm^-1^), negative PC1 bands were assigned to lignin (1605 and 1633 cm^-1^), and positive PC2 bands were assigned to silica (481 cm^-1^) and lignin monomers (1176 cm^–1^).

To study the local composition of the cell wall at the silica depositing loci, sorghum roots grown hydroponically with silicic acid were analyzed by Raman microspectroscopy. Principal Component Analysis (PCA) could identify silicified aggregates and divide them to two regions (**Figure 4**). The center, characterized by positive PC2 values, presented a major silica band at 481 cm^-1^ (Bertoluzza et al., 1982), a lignin monomers band at 1176 cm^–1^, and an aromatic carbonyl band at 1639 cm^–1^ (Bock and Gierlinger, 2019). The periphery of the aggregates, characterized by negative PC1 values, indicated high abundance of lignin bands at 1605 and 1633 cm^-1^. These bands are typical to lignin with low levels of polymerization (Diehn et al., 2024). Locations farther from the aggregate were represented by negative PC2 values, with bands typical to polysaccharides and acetylated pectin at 818, 861, and 1453 cm^-1^ (Synytsya et al., 2003; Xiao et al., 2020).

**Figure 5:**
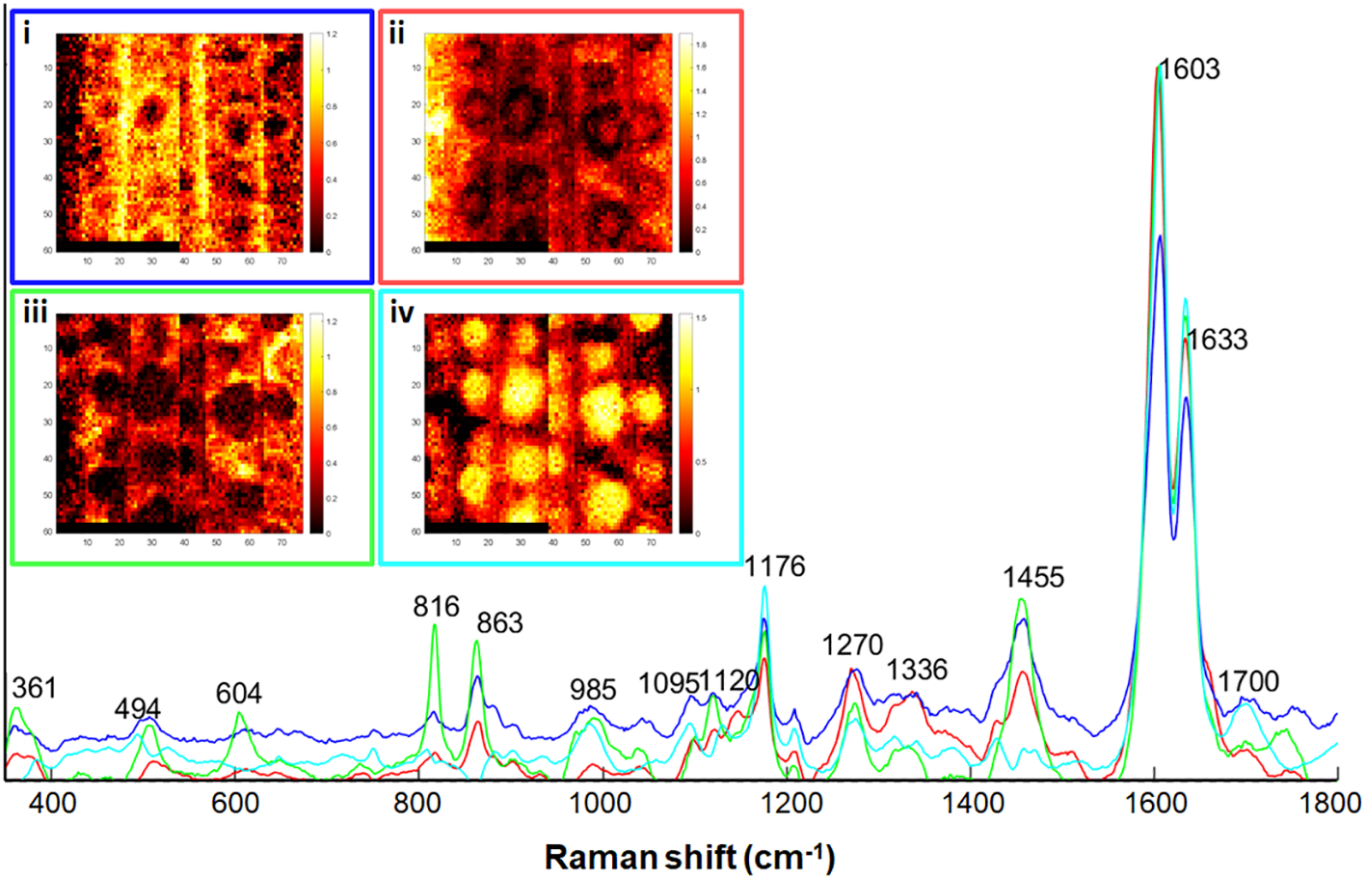
Multivariate curve resolution (MCR) analysis of two +Si maps, the right one was also analyzed by PCA in **Figure 4**. The distribution maps of each of the components is presented in the insets i-iv. Scale bars black-red to yellow-white indicates increasing component relative concentration. **(i)** Blue trace, representing the ITCW background. **(ii)** Red trace, and **(iii)** Green trace, representing non-silicified ITCW. **(iv)** Cyan trace, representing silica aggregates. See text for band assignments.

To highlight the variations within the aggregates we decomposed the spectra of the two Raman maps of +Si roots by Multivavirate Curve Resolution (MCR) analysis (**Figure 5**) using 4 components. Following the rules of Beer-Lambert law, each spectrum is described as a product of their specific pure component and a corresponding relative concentration. The first pure component (blue, **Figure 5i**) represented background ITCW, with typical bands of lignin (1176, 1270, 1600, 1633 cm^–1^), polysaccharides (816, 863, 1095, 1120 cm^-1^) aliphatic bonds (1455 cm^-1^), and silica (494, 985 cm^-1^). The second (red, **Figure 5ii**) and third (green, **Figure 5iii**) pure components represented variations in the ITCW. Both components were enriched with pectin (863 cm^-1^) and cellulose/hemicellulose (361, 816 cm^-1^). A unique band at 604 cm^–1^ could represent poly-silicic acid before its deposition into silica (Bertoluzza et al., 1982). This indicates a low concentration of lignin, and low reactivity of soluble silicic acid at the non-silicified ITCW. The fourth pure component that was strongest in the center of the aggregate (cyan, **Figure 5iv**) presented lignin monomers band at 1176 and aromatic carbonyls at 1700 cm^-1^.

**Figure 6:**
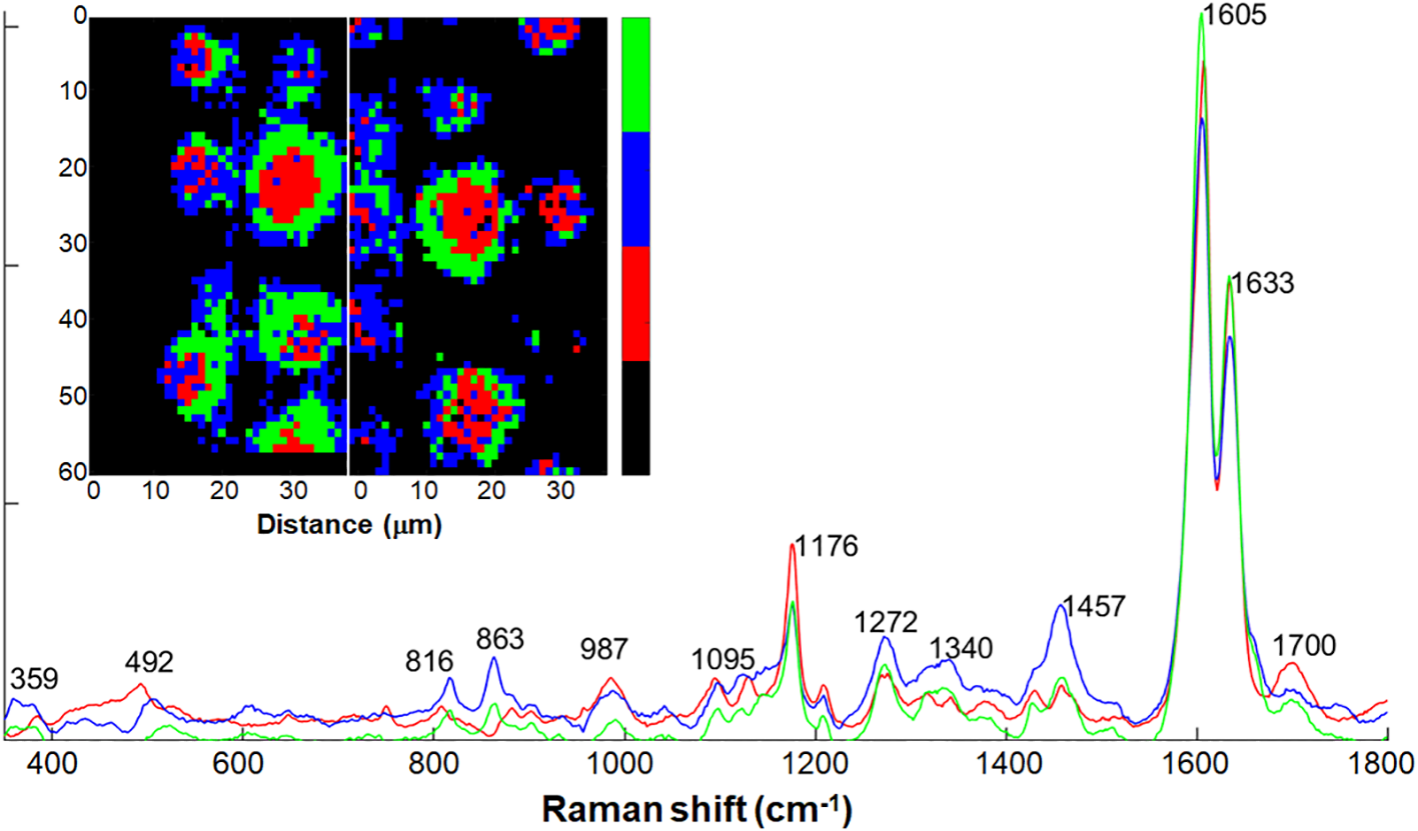
Three pure components calculated by non-negative matrix factorization (NMF) analysis on Raman spectra of intact silica aggregates selected from the maps in **Figure 5**. Two Raman maps collected from intact endodermal ITCW of +Si roots were segmented via multivariate approach, and spectra of the silica aggregates were selected (inset maps). Black indicates the non-selected spectra. The spectral dataset was clustered into three traces based on which component is the most prominent for each spectrum. Map colors indicate a pure component trace of the same color. Red regions presented at spot centers showed high abundance bands of silica at 492 and 987 cm^-1^, monolignols at 1176 cm^-1^, and aromatic carbonyl groups at 1700 cm^-1^. Blue regions presented spot periphery, with polysaccharides and pectin bands at 816, 863, 1457 cm^-1^) Green regions presented regions of chemical transition between the red and blue regions.

We successfully extracted spectra from the intact silica aggregate using Non-negative Matrix Factorization (NMF) and a threshold-based approach. Subsequently, we further clustered the extracted data using NMF to three pure components. Similarly to the PCA and MCR approaches, NMF also indicated the regions around the aggregates to contain polysaccharides and pectin, by presenting bands at 1457, 863, 816 cm^-1^ (blue component in **Figure 6**). The central regions of the aggregates presented bands typical to silica at 492 and 987 cm^-1^, and monolignols at 1176 cm^-1^ (red component in **Figure 6**). Bands at 1604 and 1633 cm^–1^ suggested low levels of lignin polymerization at the silica deposition locus. The band at 1700 cm^-1^ could indicate high abundance of aromatic esters and other carbonyl groups in the silicified lignin.

**Figure 7:**
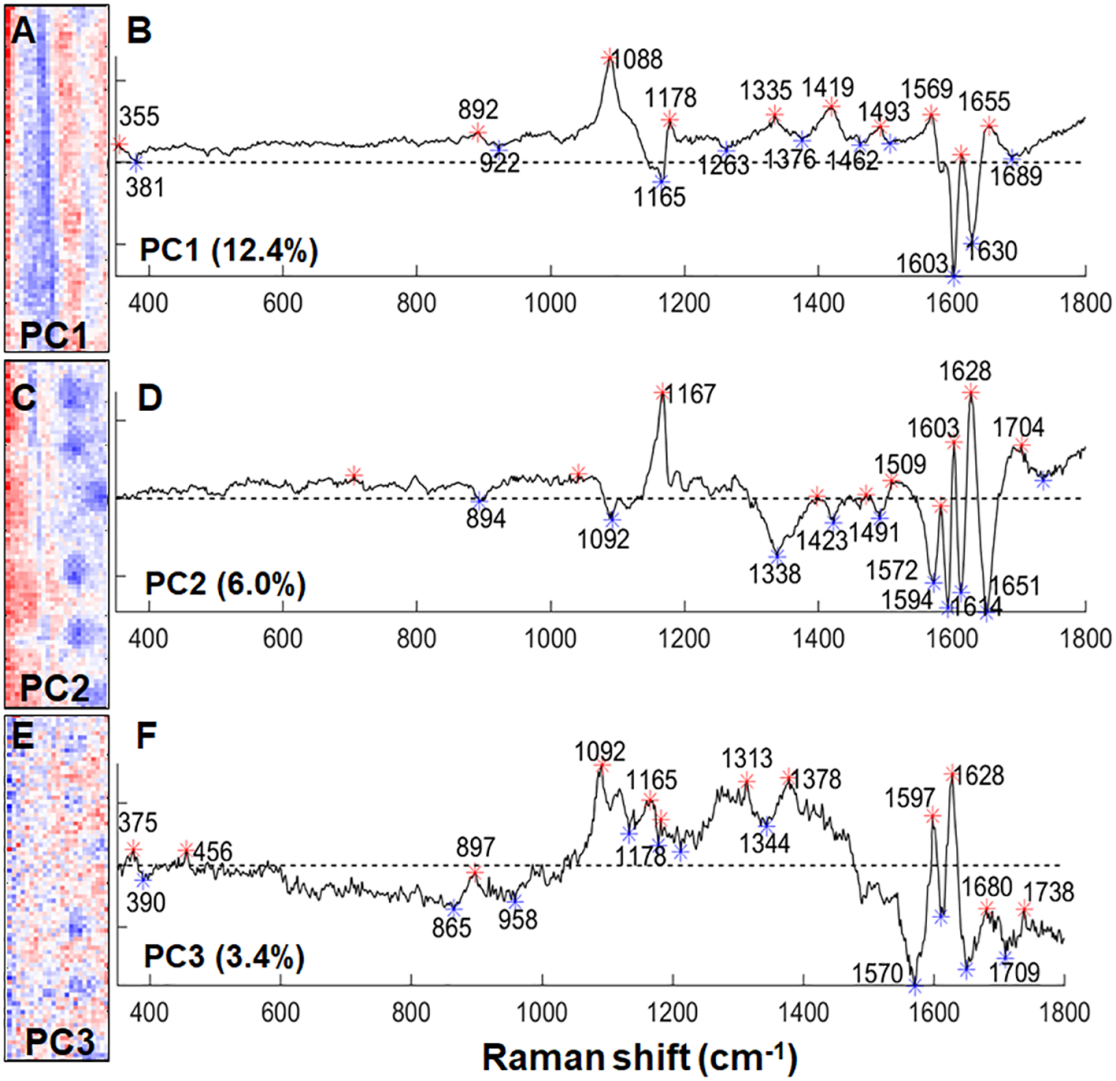
PCA on a Raman micro-spectroscopic map of the ITCW of a –Si sorghum root. **(A)** PC1 intensity map. **(B)** PC1 loading values, representing in negative bands polysaccharides (381, 1165 cm^-1^) and lignin (1603, 1630 cm^–1^). **(C)** PC2 intensity map. **(D)** PC2 loading values representing in negative values highly polymerized lignin (1600, 1614 cm^–1^) and lignin radicals (1594 cm^-1^). **(E)** PC3 intensity map. **(F)** PC3 loading values representing in positive bands polysaccharides (1092, 1165, 1313, 1378 cm^-1^) and lignin (1597, 1628 cm^–1^), and in negative bands lignin radicals (1572-1594 cm^-1^). Width of the maps is 25 μm. Asterisks on the loading graphs highlight positive (red) and negative (blue) score values.

To detect the chemical changes in lignin as a result of silica deposition, we compared the +Si Raman maps to ITCW maps of -Si roots. Similarly to our previous publications, Raman mapping highlighted the lignin spotted pattern (Zexer and Elbaum, 2020, 2022). PCA allowed the separation of the dataset to background components, represented by negative PC1, and spot components, represented by negative PC2 and PC3 (**Figure 7**). The main band in PC2 and PC3 was assigned to lignin radicals at 1572-1594 cm^-1^ (Barsberg et al., 2006). Negative loadings of PC2 at 1600 and 1614 cm^–1^ could represent highly polymerized lignin in the spot center while positive bands at 1603 and 1628 cm^-1^ possibly represent less mature lignin with a lower degree of polymerization in the background ITCW (Diehn et al., 2024). The positive values of PC3 surrounding the center of the spot represented polysaccharides at 1092, 1165, 1313, and 1378 cm^-1^ (Agarwal and Ralph, 1997).

**Figure 8:**
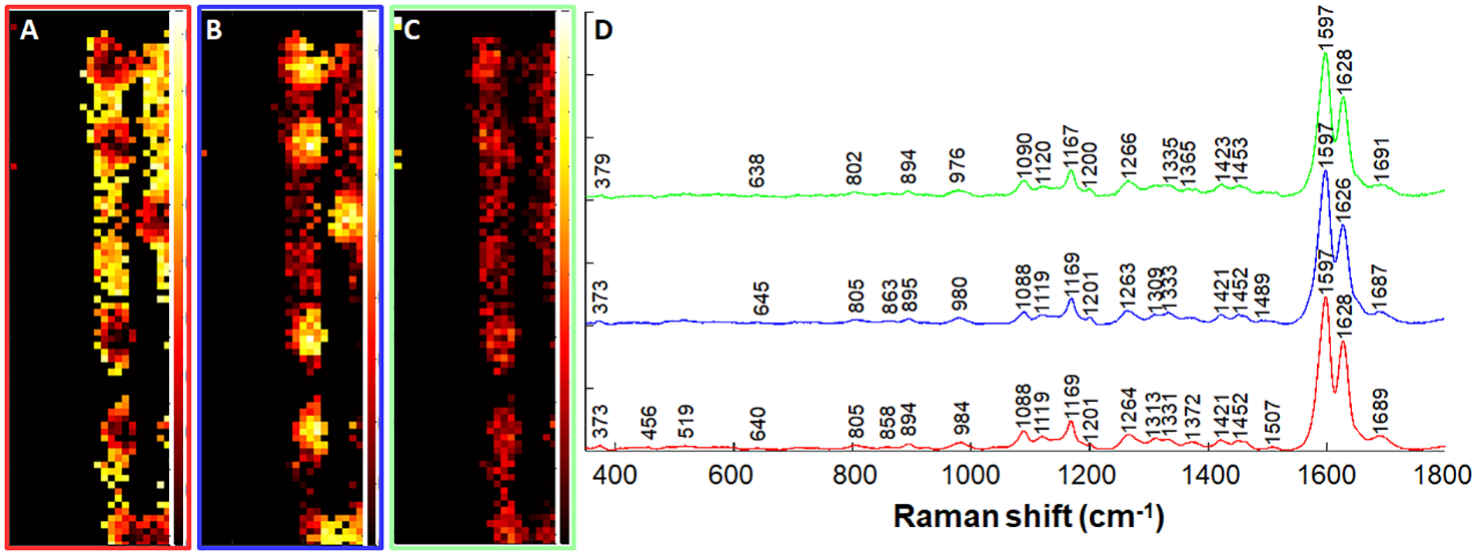
Non-negative matrix factorization (NMF) analysis of Raman spectra of intact lignin spots. A dataset of only the spots was extracted based on NMF analysis (**Figure 7**) and further segmented to three components. **(A–C)** Distribution maps of the red **(A)**, blue **(B)**, and green **(C)** components presented in panel **(D)** Black-red shading indicates low component intensity, and yellow-white shading indicates high component intensity. Map width is 25 μm. See text for band assignments.

To identify regions within the spots that may explain the variation in autofluorescence as previously reported (Zexer and Elbaum, 2020) and presented in **Figure 2**, we segmented the dataset to regions of the lignin spots separately from the rest of the ITCW through NMF analysis. Spectra of the spots were extracted and further grouped according to three NMF pure components (**Figure 8**). We could define gradients of decreasing intensity from the periphery of the spot (component 1) to its center (component 2) in bands at 1689 cm^–1^ (aromatic carbonyls), 1507 cm^-1^ (lignin skeleton (Bock et al., 2020), and 1313 cm^-1^ (xylan (Agarwal, 2014), in relation to aliphatic hydroxyls, represented by the 1331 cm^-1^ band (Agarwal, 2019).

## Conclusions

Silica aggregation onto lignified loci in the ITCW of sorghum roots presents a simple bio-silicification system in plants, which can be readily observed by microscopy. A unique monolignol composition is extruded and polymerized locally into the forming ITCW. This results in radial gradients of cell wall compounds that are initiated at the extrusion locus. Local concentration of oxidating enzymes that bind and consume monolignols may limit their spreading, as was shown for tracheary elements in Arabidopsis primary roots (Perkins et al., 2022). Raman microspectroscopy could show that monolignols were trapped in the forming silica, and the extent of lignin polymerization was limited. Reduction in lignin polymer length is typical to synthetic lignin which is polymerized in the presence of silicic acid (Radotić et al., 2022). Such reduction suggests inhibited formation β-O-4 ether linkage, which is the most prevalent bond between monolignols (Boerjan et al., 2003). Similar processes may govern the biological process as well, possibly by the binding of phenolic hydroxyls to Si-OH in silicic acid molecules thereby jeopardizing the formation of β-O–4 linkages in the deposited lignin.

When no silicic acid was available, the monomers possibly diffused to some distance and then formed highly polymerized lignin that crosslinked the cell wall. This could explain the appearance of dense cell wall shaped as doughnuts at the non-silicified ITCW. High carbonyls abundance was detected in both silicified and non-silicified wall. We therefore conclude that other lignin groups interacted with silicic acid. The carbonyls could belong to ferulic acid moieties and play roles in the autofluorescence shifts. In the absence of silicic acid, it could crosslink the lignin and hemicellulose (Hatfield et al., 2017). In the presence of silicic acid, the competition on the β-O-4 position with silicic acid may push ferulic acid to forming ester bonding with hemicellulose (Ishii, 1997). Further chemical analysis at the microscopic range is needed to understand plant bio-silicification.

## Materials and methods

### Plant material and growing conditions

Grains of *Sorghum bicolor* (L.) Moench, line BTx623 were surface-sterilized with 3% sodium hypochlorite for 10 min and washed twice with distilled water. Grains were then placed in petri dishes lined with wet filter papers and germinated for 72 h. After germination, seedlings were grown hydroponically for one week. All growth solutions were based on double distilled water containing 1 mM CaCl_2_. Si+ medium was supplied with sodium silicate (Na_2_SiO_3_) at a final concentration of 2 mM. Si- medium was provided with NaCl at a similar final concentration in order to maintain equal ionic balance across all media. The final pH of all the solutions was adjusted to 5.8 with HCl. Seedling were grown on plates of polystyrene foam mounted on top darkened 1L beakers containing growing media. In each beaker we grew ten seedlings. Cultivation was done in a growth chamber, under controlled conditions with photoperiod 16 h: 8 h (light: dark) illuminated with photosynthetic active radiation (PAR) of approximately 200 μmol m^-2^ s^-1^, 28°C: 22°C (light: dark) temperature, and 70% air humidity.

### Root sectioning

Complete primary roots were harvested and fixed in ethanol: acetic acid (9:1 v/v) under vacuum for 24 hours. Root samples were kept in the same fixative at 4°C for additional 48 hours. The fixing solution was then replaced with ethanol 70% and samples were stored at 4°C until use. Root sections of 4 cm, 12 – 16 cm from root tip were carefully peeled out of their outer cortical tissues using finely serrated tweezers.

### Confocal and fluorescence microscopy

Root sections of 20 mm in length were mounted on glass microscope slides. Samples were immersed in double distilled water and covered with covering slips. Observations and images were made using a Leica SP8 Confocal Laser Scanning Microscope equipped with an X63 water immersed objective. Excitation wavelengths used were 405 nm and 488 nm; emission was collected at 400-500 nm and 500-550 nm respectively.

### Raman micro-spectroscopic observation of endodermal cell wall

Peeled root sections were mounted on aluminum microscope slides using super glue. The samples were covered with a drop of double distilled water and measurements were made using an X63 water immersed objective. Raman maps were collected with a Renishaw InVia spectrometer equipped with 532 nm laser (45 mW max. intensity, 2 μm^2^ beam), utilizing WiRE3.2 software (Renishaw, New Mills, UK). Measurements were performed using the Streamline mode with acquisition time of 30 seconds, resulting in maps with a spot size of 1 µm in x and y direction.

### Raman data processing and analysis

Raman maps were pre-processed and analyzed using Matlab R2020b (The MathWorks, Inc, Natick, MA, USA). Data pre-processing steps included the data managing with sorting the spectra and eventually extracting selective spectra corresponding to specific spots or tissues using approaches for multivariate classification (Liedtke et al., 2021). Spectra were interpolated with a distance of 1.8 cm^-1^ in the range of 350 to 1800 cm-^1^ in order to assure equal distances between data points. Subsequently, the background in the spectra were removed by applying asymmetric least square (AsLS) correction as proposed by Eilers (Eilers, 2003) and finally the spectra were vector-normalized. Chemical mapping and multivariate images analysis were carried out using Principal Component Analysis (PCA), Non-negative Matrix Factorization (NMF) and Multivariate Curve Resolution (MCR) (Juan et al., 2014). Latter was executed using Hypertool3 (Amigo et al., 2015).

## Data availability statement

The raw data supporting the conclusions of this article will be made available by the authors, without undue reservation.

## Author contributions

RE: Conceptualization, Funding acquisition, Supervision, Writing – original draft, Writing – review & editing. NZ: Conceptualization, Data curation, Formal analysis, Visualization, Writing – review & editing. SD: Conceptualization, Data curation, Formal analysis, Visualization, Writing – review & editing.

## Supplementary material

Supplementary Video 1 Confocal fluorescence microscopy of the ITCW of a +Si root. Silica aggregates are seen in blue autofluorescence and the non-silicified cell wall appears in green.

Supplementary Video 2 Confocal fluorescence microscopy of the ITCW of a -Si root. The locations that are predicted to silicify autofluorescence in green while the ITCW at the periphery of the predicted silicification locus appears in blue.
